# Use of Complementary and Alternative Medicine in Children with Cancer: A Study at a Swiss University Hospital

**DOI:** 10.1371/journal.pone.0145787

**Published:** 2015-12-22

**Authors:** Tatjana Magi, Claudia E. Kuehni, Loredana Torchetti, Laura Wengenroth, Sonja Lüer, Martin Frei-Erb

**Affiliations:** 1 Swiss Childhood Cancer Registry, Institute of Social and Preventive Medicine, University of Bern, Bern, Switzerland; 2 Institute of Complementary Medicine, University of Bern, Bern, Switzerland; 3 Division of Pediatric Hematology/Oncology, University Children’s Hospital Bern, Inselspital, Bern, Switzerland; Yong Loo Lin School of Medicine, National University of Singapore, SINGAPORE

## Abstract

**Background:**

Though complementary and alternative medicine (CAM) are frequently used by children and adolescents with cancer, there is little information on how and why they use it. This study examined prevalence and methods of CAM, the therapists who applied it, reasons for and against using CAM and its perceived effectiveness. Parent-perceived communication was also evaluated. Parents were asked if medical staff provided information on CAM to patients, if parents reported use of CAM to physicians, and what attitude they thought physicians had toward CAM.

**Study Design:**

All childhood cancer patients treated at the University Children’s Hospital Bern between 2002–2011 were retrospectively surveyed about their use of CAM.

**Results:**

Data was collected from 133 patients (response rate: 52%). Of those, 53% had used CAM (mostly classical homeopathy) and 25% of patients received information about CAM from medical staff. Those diagnosed more recently were more likely to be informed about CAM options. The most frequent reason for choosing CAM was that parents thought it would improve the patient’s general condition. The most frequent reason for not using CAM was lack of information. Of those who used CAM, 87% perceived positive effects.

**Conclusions:**

Since many pediatric oncology patients use CAM, patients’ needs should be addressed by open communication between families, treating oncologists and CAM therapists, which will allow parents to make informed and safe choices about using CAM.

## Introduction

Parents often choose complementary and alternative medicine (CAM) to supplement treatment of children and adolescents with cancer [1–3]. In a recent systematic review, the prevalence of CAM use for patients with pediatric cancer ranged from 6%-91% [4], varying on a country-by-country basis. Homeopathy and dietary supplements are most common in Germany [5], while water therapy and Spirulina are popular in Malaysia, and herbal extracts in Mexico [6,7]. Patients use a broad spectrum of CAM methods, and some may interact with conventional medicine, causing adverse effects [8–12]. In Switzerland CAM is very popular: a 2007 study showed 23% of the adult Swiss population had used CAM within the past year [13]. Zuzak et al. reported that 58% of patients who presented at a Swiss pediatric emergency department had used CAM, but as yet, there is no data on how widespread CAM use is in pediatric oncology [14]. Pediatric oncology patients often use CAM without telling the oncologists who treat them [1,3,7,15,16].

Since 2010, a collaboration between the Division of Pediatric Hematology/Oncology at the University Children’s Hospital and the Institute of Complementary Medicine (IKOM), both at the University of Bern, exists. Parents of children with cancer who are hospitalized for the first time receive detailed written information about the clinic including a sheet about CAM offers of the IKOM. On request, families who want further information about CAM are referred by the pediatric oncologist to the IKOM for counseling and/or complementary treatment. Those patients are followed by the IKOM medical staff once a week during hospital stays, and on demand the IKOM staff provides outpatient care.

This study was designed to collect information on CAM use by childhood cancer patients, focusing particularly on the following: (1) the overall proportion of patients who use CAM, CAM methods applied, and the therapists who provide CAM; (2) the reasons parents did or did not choose CAM; (3) communication between parents and doctors about CAM, any change over the past decade in the proportion of patients who were informed about CAM by medical staff, and physicians’ attitudes regarding their patients’ use of CAM; and, (4) parent and/or patient perceptions of the effectiveness of CAM.

## Methods

### Study Population

The study was carried out in collaboration with the Swiss Childhood Cancer Registry (SCCR, www.childhoodcancerregistry.ch), which, since 1976, has included all children and adolescents in Switzerland diagnosed with leukemia, lymphoma, central nervous system (CNS) tumors, malignant solid tumors and Langerhans cell histiocytosis (LCH) before they were 21 [17]. This study included all patients registered at the SCCR who were diagnosed between January 1^st^ 2002 and December 31^st^ 2011, were aged 0 to 18 years, and were treated at the Division of Pediatric Hematology/Oncology at the University Children’s Hospital Bern, Switzerland. Exclusion criteria were death within 2 months of diagnosis, and the parents’ refusal to participate in a questionnaire survey.

The SCCR was granted ethical approval through the general cancer registry permission (issued by the Swiss Federal Commission of Experts for Professional Secrecy in Medical Research) and a non obstat statement was obtained from the ethics committee of the canton of Bern. For the survey on CAM use, ethical approval was not necessary at the time of the study conduction. Written informed consent was obtained by the participants.

### Questionnaire Survey

A German language questionnaire on CAM use was designed for the parents of children with cancer. It was based on published international studies and specifications of CAM use in Switzerland [1–3,15,16,18,19]. There were a total of 18 questions: six questions about socio-demographic background and conventional cancer treatment, followed by 12 questions about CAM use.

The questionnaire was tested for comprehensibility by 12 people (six healthy adults, four physicians and two adolescent patients). On May 29^th^ 2012, the questionnaires were mailed to families with a cover letter, detailed study information and an informed consent form. Patients who did not return the informed consent and questionnaire within 6 weeks received a postal reminder. Along with the reminder, families who lived in the French speaking part of Switzerland received a second questionnaire in French.

### Definition and Assessment of CAM Use

There is no generally accepted definition of CAM [20], but the one most commonly used was issued by the U. S. National Center for Complementary and Integrative Health (NCCIH). NCCAM defines CAM as a group of diverse medical and health care systems, practices, and products not generally considered part of conventional medicine [21]. CAM practices are grouped into three categories: natural products; mind-body medicine; and, manipulative body-based practices.

In our questionnaire, we assessed the way families used CAM before and after the cancer diagnosis, and asked which therapies they had used. Respondents could select from 43 listed CAM therapies and treatments, and could have reported additional methods. CAM methods were grouped into six categories for this analysis: medicaments and remedies (e.g., homeopathy, traditional Chinese medicine, Ayurveda); regulatory therapies (e.g., acupuncture, shiatsu); nutrition (e.g., dietary supplements, juice diet); mind-body therapies (e.g., yoga, music therapy); manual therapies (e.g., massages, chiropractic); and, other therapies (i.e., bioresonance therapy, electromagnetic therapy). We asked who provided these therapies (e.g., physician, non-medical practitioner or therapist or parents), whether CAM was used in a complementary fashion, or as an alternative to conventional treatment, and if CAM users would recommend that therapy to other patients and families. CAM users and non-CAM users were asked why they did or did not use CAM. CAM users were also asked if they had told the treating oncologist, their primary care physician, or pediatrician about the additional therapies they had used, and, if so, what the doctor´s reaction had been. Families were also asked if medical staff had informed them about CAM therapies when they discussed cancer treatment, and if they would have wanted to receive this information from their treating physician. The survey also asked if CAM users thought the therapies they tried were effective (positive or negative effect, or both) and asked them to specify the effects they attributed to CAM therapy.

### Assessment of Socio-demographic and Cancer-related Data

This study extracted baseline demographic data and prospectively collected medical information on diagnosis and treatment of patients from the SCCR, including age, gender, cancer diagnosis, age at diagnosis, cancer treatment, relapse, and time since diagnosis. The International Classification of Childhood Cancer, 3rd Edition (ICCC-3) was used to classify diagnoses [22]. Treatment was classified as surgical tumor resection (yes/no), chemotherapy (CTX; yes/no), radiotherapy (RTX; none, body and limb irradiation, cranial and spinal irradiation), and bone marrow or peripheral blood stem cell transplantation.

### Statistical Analysis

Results are presented as descriptive statistics, with percentages and 95% confidence intervals (95% CI). Characteristics of participants included into the analysis and of non-participants were compared with chi-square-tests (gender, deceased at time of study, diagnoses) and *t*-test (age), accordingly. The Cochran-Armitage test for trend was used to assess if the proportion of patients told about CAM by medical staff had increased over the last decade. Data were analyzed with the Statistical Package for Social Sciences (IBM SPSS, version 21.0, IBM Corp., Armonk, NY).

## Results

### Characteristics of the Study Population

**Fig 1:**
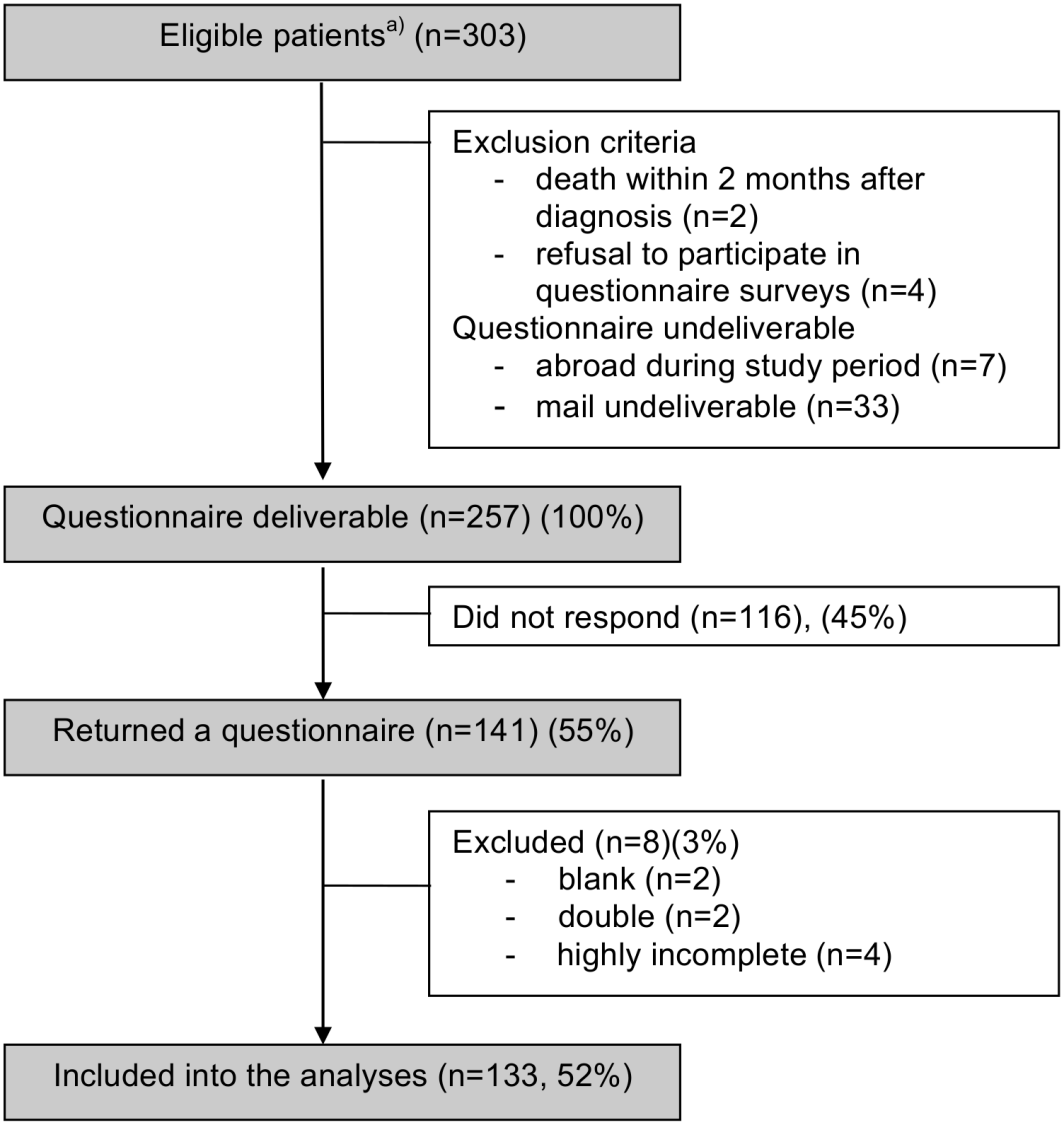
Inclusion process and eligibility of study participants. ^a)^ Diagnosed between January 1^st^ 2002 and December 31^st^ 2011, aged 0 to 18 years at diagnosis, treated at the Division of Pediatric Hematology/Oncology at the University Children’s Hospital Bern, Switzerland.

Of 303 eligible patients, 46 were excluded due to the exclusion criteria or because their mail was undeliverable (Fig 1). A total of 257 families were contacted. Of these, 141 (55%) returned the questionnaire. Eight questionnaires (3%) were excluded leaving 133 questionnaires (52%) in the final analyses. Children from participating families did not differ from children of non-participants regarding sex, age, and diagnosis (all p-values > 0.14; S1 Table). The only difference was a higher proportion of deceased children (25% vs. 11%, p < 0.01) in non-participants.

Of the 133 participants included mean age at diagnosis was 6.6 years (SD = 4.9; Table 1); 63 (47%) were girls. By the time the survey was mailed, 15 patients (11%) had died. The most common diagnoses were leukemia (31%), CNS neoplasms (24%) and lymphomas (16%). Most children (93%) had finished conventional cancer treatment, which consisted of CTX (83%), RTX (29%), and surgery (44%). Most parents had completed vocational training or upper secondary education (77% mothers; 62% fathers). Most families had a monthly family-income between CHF 5.000 and 10.000 (64%). Mothers completed the majority of questionnaires (90%).

**Table 1:** Characteristics of the Participants included into the Analysis (N = 133).

Variables	Number (percentage)
**Clinical information**	133 (100)
*Age at diagnosis*	
0–4 years	60 (45)
5–9 years	29 (22)
10–13 years	28 (21)
14–18 years	16 (12)
*Gender*	
Female	63 (47)
Male	70 (53)
*Deceased at time of study*	15 (11)
*Diagnoses (ICCC-3 main group)*	
(1) Leukemia, myeloproliferative diseases and myelodysplastic syndrome	41 (31)
(2) Lymphoma and reticuloendothelial neoplasms	21 (16)
(3) CNS tumors and miscellaneous intracranial and intraspinal neoplasms	32 (24)
(4) Neuroblastoma and other peripheral nervous cell tumors	9 (7)
(8) Malignant bone tumors	7 (5)
(9) Soft tissue and other extraosseous sarcomas	8 (6)
Other diagnoses ^a)^	15 (11)
*Conventional treatment* ^b)^	
CTX	110 (83)
Surgery	59 (44)
RTX	38 (29)
Blood stem cell transplantation	15 (11)
*Treatment status*	
On treatment at time of study	7 (5)
Treatment finished at time of study	124 (93)
Missing answer	2 (2)
**Sociodemographic information**	
*Monthly family-income (n = 121)*	
< CHF 5.000	17 (14)
CHF 5.000–10.000	77 (64)
> CHF 10.000	27 (22)
*Mothers’ education*	
Compulsory school	9 (7)
Vocational training/upper secondary education	103 (77)
University	18 (14)
Missing answer	3 (2)
*Fathers’ education*	
Compulsory school	8 (6)
Vocational training/upper secondary education	83 (62)
University	37 (28)
Missing answer	5 (4)
*Respondent* ^b)^	
Mother	119 (90)
Father	34 (26)
Child	9 (7)
Others	1 (1)

ICCC-3, International Classification of Childhood Cancer, 3rd Edition; CNS, central nervous system; CTX, chemotherapy; RTX, radiotherapy.

^a)^ Including ICCC-3 main groups: (6), (7), (10), (11) and (14);

^b)^ Multiple answers possible.

### Use of CAM

About half of the participating families (n = 70, 53%) reported that they used CAM before their child was diagnosed with cancer. Of these families, 54 (77%) continued to use CAM after the cancer diagnosis, and 17/63 families who had not previously used CAM (24%) began to use it. Of participating patients, 53% (n = 71) reported they used CAM after the cancer diagnosis (Table 2). Among CAM users, the most common methods applied after diagnosis were classical homeopathy (54%), dietary supplements (31%), prayer/faith (30%), and over-the-counter homeopathy (27%).

**Table 2:** Usage of CAM in Childhood Cancer Patients.

		CAM users after cancer diagnosis (n = 71)		All patients (N = 133)	
Overall use of CAM	Number	(%)	95% CI^a)^	(%)	95% CI^a)^
CAM usage after the cancer diagnosis	71	-		(53)	44–62
No CAM usage after the cancer diagnosis	62	-		(47)	38–56
*Number of CAM methods used*	71	(100)		(53)	
1 method used	19	(27)	16–37	(15)	9–20
2 methods used	13	(18)	10–28	(10)	5–15
3–4 methods used	15	(21)	12–30	(11)	6–17
>4 methods used	24	(34)	23–45	(18)	12–25
*CAM use*					
Complementary	63	(89)	86–98	(47)	40–57
Alternative	4	(6)	1–12	(3)	1–7
Both	1	(1)	0–5	(1)	0–2
Missing	3	(4)		(2)	
*Drugs and remedies (at least one method)* ^b)^:	59	(83)	74–91	(44)	36–53
Classical Homeopathy	38	(54)	41–65	(29)	21–37
Over the counter Homeopathy	19	(27)	17–37	(14)	9–21
Bach flowers	16	(23)	14–32	(12)	8–17
Spagyric	16	(23)	13–32	(12)	7–17
Schüssler Salts	15	(21)	12–31	(11)	6–17
Anthroposophic medicine	8	(11)	4–20	(6)	2–11
Herbal medicaments	6	(9)	3–15	(5)	2–8
Mistletoe therapy	5	(7)	1–13	(4)	0–7
Aromatherapy	3	(4)	0–10	(2)	0–5
Traditional Chinese medicine	2	(3)	0–8	(2)	0–4
Ayurveda	1	(1)	0–5	(1)	0–2
*Regulatory therapies (at least one method)* ^b)^:	18	(25)	16–36	(14)	8–20
Kinesiology	12	(17)	9–27	(9)	5–14
Acupuncture	5	(7)	2–14	(4)	1–8
Meridian therapy	3	(4)	0–10	(2)	0–5
Shiatsu	1	(1)	0–5	(1)	0–2
*Nutrition (at least one method)* ^b)^:	27	(38)	27–49	(20)	14–27
Dietary supplements	22	(31)	20–42	(17)	11–23
Special dietary change	7	(10)	4–18	(5)	2–10
Fasting or juice cure	2	(3)	0–8	(2)	0–4
Macrobiotics	1	(1)	0–5	(1)	0–2
*Mind-body therapies (at least one method)* ^b)^:	29	(41)	30–53	(22)	15–29
Prayer/Faith	21	(30)	19–41	(16)	10–22
Music therapy	11	(16)	7–24	(8)	4–13
Art therapy	4	(6)	1–12	(3)	1–7
Meditation	3	(4)	0–10	(2)	0–5
Autogenic training	1	(1)	0–5	(1)	0–2
Breath therapy	1	(1)	0–5	(1)	0–2
Biofeedback	1	(1)	0–5	(1)	0–2
*Manual therapies (at least one method)* ^b)^:	23	(32)	22–43	(17)	11–24
Osteopathy	9	(13)	5–20	(7)	3–11
Massage	7	(10)	3–18	(5)	2–10
Reflexology	6	(9)	2–16	(5)	2–8
Cranio-sacral therapy	3	(4)	0–9	(2)	0–5
Lymphatic drainage	1	(1)	0–5	(1)	0–2
Chiropractic	1	(1)	0–5	(1)	0–2
Acupressure	1	(1)	0–5	(1)	0–2
*Others (at least one method)* ^b)^:	19	(27)	17–36	(14)	9–21
Bioresonance therapy	10	(14)	6–24	(8)	4–13
Electromagnetic therapy	2	(3)	0–8	(2)	0–4
Other methods	15	(21)	12–31	(11)	6–17

CAM, Complementary and alternative medicine; CI, confidence intervals.

^a)^Bootstrapped CI for percentage;

^b)^Multiple answers possible.

About half of CAM users (n = 32, 45%) used one or two methods, and the other half (n = 39, 55%) used more than two methods. Almost all CAM users (n = 63, 89%) reported that their CAM use complemented conventional treatment. For palliative therapy, four respondents (6%) used only CAM (no conventional treatment). One family reported that when their child entered a palliative phase, they stopped using CAM as a complement and began using it as an alternative treatment.

The therapists who treated patients with CAM were mostly non-medical practitioners (47%), physicians (41%), and parents (41%; Table 3). In 56% (n = 40) of cases, no physician was involved in CAM therapy.

**Table 3:** Information on the Therapist applying CAM, and parent-reported Reasons for and against CAM Use.

**Applying therapist** ^a)^	Number (percentage)
Non-medical practitioner ^b)^	33 (47)
Physician	29 (41)
Parents	29 (41)
Pharmacist	10 (14)
Non-medical therapist ^c)^	14 (20)
Missing	2 (3)
**Reasons for CAM use (n = 71)** ^a)^	Number (percentage)
To improve general condition	53 (75)
To strengthen the immune system	47 (66)
To reduce side effects of conventional treatment	42 (60)
To improve the chance of cure	35 (49)
For relaxation	34 (48)
Trying all possible therapies	15 (21)
Other reasons	3 (4)
**Reasons for no CAM use (n = 62)** ^a)^	Number (percentage)
Lacking information on CAM	19 (31)
Belief that CAM is ineffective	19 (31)
To avoid further stress	17 (27)
Fear of interactions with conventional treatment	10 (16)
Adequate efficacy and/or good tolerance of conventional treatment	10 (16)
Oncologist discouraged from CAM	7 (11)
Fear of side effects of CAM	2 (3)
Family doctor discouraged CAM use	2 (3)
Other reasons	3 (5)

CAM, complementary and alternative medicine.

^a)^ Respondents could indicate more than one answer.

^b)^ Non-medical practitioners apply acupuncture, homeopathy or naturopathy.

^c)^ Non-medical therapists apply different non-interventional therapies like e.g., kinesiology, massage.

### Reasons for or against Choosing CAM

The most important reasons for using CAM were a wish to improve the patient’s general condition (75%), to strengthen their immune system (66%), and to reduce adverse effects of conventional treatment (60%; Table 3). The main reasons parents did not choose CAM were not enough information about CAM (31%), the belief that CAM is ineffective (31%), and desire to avoid putting the child under more stress (27%).

### Information and Communication about CAM

Most parents (n = 53, 75%) reported that they told treating oncologists they were using CAM to treat their children. Over half the participating families had not been told that CAM was available (n = 79, 59%), 33 (25%) were informed; and 21 (16%) could not remember. About half of the participants (n = 71, 53%) noted that they would have appreciated information about possible treatment with CAM, while 47 (35%) would not have wanted this information, and 15 (11%) did not answer the question. Over the studied years of diagnoses (2002–2011), the proportion of families who learned about CAM from medical staff increased (p_trend_ = 0.034). Of families with children diagnosed 2002–2006 17% were informed about CAM by hospital staff. In 2007–2011, this increased to 32% of families with children diagnosed. Most CAM users learned about CAM from friends (n = 32, 45%) and family (n = 22, 31%), other unspecified sources (n = 18, 25%), family doctors/pediatricians (n = 17, 24%), media (n = 16, 23%), other parents (n = 12, 17%), oncologists (n = 8, 11%), or nonmedical practitioners (n = 8, 11%). Family doctors and pediatricians were more supportive of CAM use than were pediatric oncologists (Table 4).

**Table 4:** Physicians informed by the Families about CAM Use and their Reactions (CAM users; n = 71).

*Physicians informed* ^a)^	Pediatric oncologists: number (percentage)	Family doctors/ pediatricians: number (percentage)	Other doctors: number (percentage)
Yes	53 (75)	37 (52)	6 (8)
No	16 (23)	32 (45)	63 (89)
Answer missing	2 (3)	2 (3)	2 (3)
*Physicians’ reactions* ^b)^			
Agreed with CAM use	15 (28)	21 (57)	3 (50)
Took note of CAM use	26 (49)	14 (38)	3 (50)
Warned against CAM	9 (17)	1 (3)	-
Reaction not remembered	2 (4)	-	-
Answer missing	3 (6)	3 (8)	-

CAM, complementary and alternative medicine.

^a)^ Multiple physicians could be informed.

^b)^ Percentages refer to the respective number of physicians informed.

### Perceived Effectiveness

**Fig 2:**
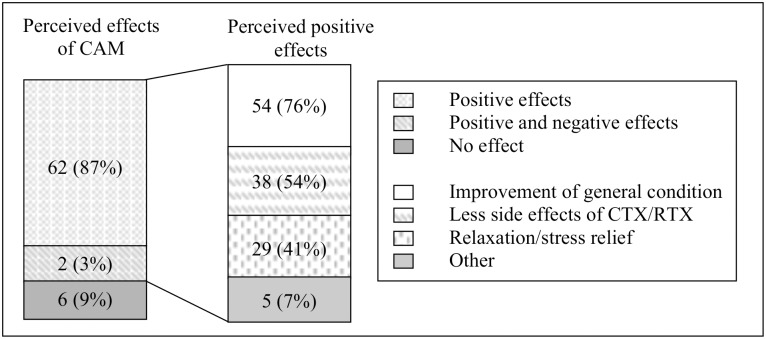
Perceived effects of CAM by the 71 users (100%; one answer missing). CAM, complementary and alternative medicine; CTX, chemotherapy; RTX, radiotherapy. Respondents could indicate more than one positive effect.

Most CAM users thought the CAM methods they used had positive effects (n = 62, 87%). A belief that the patient’s general condition had improved was most common (n = 54, 76%; Fig 2).

Almost all CAM users would recommend that other families try the methods they applied (n = 64, 90%). The main reason parents would not recommend CAM (n = 3, 4%) is that they believe that the choice to use CAM or not is an individual one, to be made by each patient/family (missing n = 4, 6%).

## Discussion

Over half (53%) of survey responders treated at this tertiary care pediatric oncology center used CAM, most commonly in the form of classical homeopathy. CAM added to conventional cancer treatments, not a substitute for them. A physician was involved in CAM therapy for less than half (41%) of patients. Mainly patients were treated by non-medical practitioners or by parents. The main reason parents gave for using CAM was to improve the patient’s general health. Important reasons for not using CAM were lack of information (31%), or a belief that CAM is ineffective (31%). Most parents (75%) told their pediatric oncologist that they were using CAM. Over the past ten years, the proportion of patients told about CAM by medical oncology staff increased. Family doctors and pediatricians were more supportive of CAM than pediatric oncologists. The majority of CAM users (87%) thought that CAM therapy had helped.

### Comparison with other studies and interpretation of results

This pediatric cancer patients from our Swiss study used CAM more often than pediatric cancer patients in other European countries [1,2,15]. But findings are in line with a recent survey conducted at a Swiss pediatric emergency department, where 58% of patients had used or were currently using CAM [14]. As in other studies from European countries, the most common CAM therapies were homeopathy and over-the-counter medications [2,5,15]. Though a survey from Germany found that anthroposophical medicine (including mistletoe therapy) was commonly used [5], this was not the case in Switzerland.

The systematic review by Bishop et al. found that most parents chose CAM to support conventional treatment, cure or fight the child’s cancer, and provide symptomatic relief [4], and the results from this study are similar. Most parents (75%) wanted to improve the child’s general condition (75%), strengthen the immune system (66%), and improve their chances of cure (49%). But this study also evaluated the reasons parents did not use CAM [2–4,6,7,15,16]. As in studies by Längler et al. and Fernandez et al., this study found that a major reason why parents did not choose CAM as an add-on therapy was lack of information [1,23]. Another reason, not mentioned so far in the literature, is that many parents thought CAM was unnecessary since the patient tolerated conventional treatment well and it was effective.

Like parents in Germany, most parents in Switzerland told treating physicians they were using CAM [1]. A study from Mexico found that only 9% of CAM users informed their physicians [7]. CAM might be less accepted by both the general population and physicians in Mexico. In Switzerland, parents reported that family doctors were usually supportive, but most pediatric oncologists simply noted CAM usage, rather than taking a position for or against it. It is possible that pediatric oncologists do not have in-depth knowledge about CAM therapies. A U.S. study found that only 43% of pediatric oncologists asked their patients if they used CAM; the main reason they did not ask was that they knew little about CAM methods (93%) [24]. This study found that over half of pediatric oncologists would like to learn more about CAM methods [24]. The majority (99%) believed that it is important to know about CAM in order to prevent harmful drug interactions between CAM and conventional cancer treatment.

A Dutch study had similar results to this one: 88% of all respondents would have liked it if their treating physician had given them information on CAM treatment; 64% of the parents would have liked it if CAM methods had been offered in the hospital [15]. A survey in Israel showed that childhood cancer patients would have liked to receive CAM therapy at the same hospital where they were conventionally treated [16].

In families with a child diagnosed more recently (2007–2011), a higher proportion of parents were told about CAM methods than in families where the child had been diagnosed earlier (2002–2006). Perhaps pediatric oncologists are now more aware of their patients’ use of CAM and understand there is a need for open communication on the topic. Oncologists may be more aware because discussion of CAM has increased at the pediatric oncology unit, due to staff members becoming more aware of different CAM methods, or because CAM physicians from the IKOM were regularly consulted in the department. Referred patients who decided to add CAM therapies to conventional cancer treatments were visited by the IKOM medical staff once a week while they were hospitalized. This fostered direct communication between pediatric oncology and IKOM staff.

From our findings that CAM was applied mainly by non-medical therapists and as self-medication by the parents arises some questions about i) the ability of non-medical therapists in the treatment of children with cancer; ii) how parents choose and prescribe CAM-medicaments and iii) a possible difference in the benefit of CAM used in combination with conventional therapy prescribed and applied by CAM-physicians, non-medical therapists or self-medication. To our knowledge there are no investigations about these topics.

Like a recent study from the Netherlands, where 49% of the patients thought CAM was very effective, and 26% thought it somewhat effective, this study found that most parents believed CAM was effective (87%) [15]. Most parents (90%) would recommend CAM to other families.

### Methodological considerations

The response rate of 55% was acceptable. Only 8 questionnaires (3%) had to be excluded, which suggests that most participants understood the questions. Socio-demographic and clinical characteristics did not differ between participants included into the analysis and non-participants, except for the survival rate, which was higher in participants. Therefore it is unclear, if the data presented for parents of children who died are fully representative for this group. Because of the survey’s retrospective design, memory bias might have influenced the outcome. Since this study was carried out in a single pediatric cancer center, it might not represent all of Switzerland. We cannot exclude sampling bias, possibly by a higher response rate of families interested in CAM or by more educated individuals.

## Conclusions

Further study is required to determine what patients, pediatric oncologists and other healthcare professionals need to know about CAM use, and its risks and benefits in treating childhood cancer. For patient safety and best care, pediatric oncologists and primary care physicians should learn more about CAM methods, and CAM providers should learn about conventional cancer treatments. Physicians should ask their patients if they use any form of CAM and advise users to cooperate with qualified CAM physicians. Coordination and cooperation between pediatric oncologists and CAM physicians could improve patient care by opening communication, increasing mutual understanding, increasing patient satisfaction, compliance and, ultimately, safety.

## Supporting Information

S1 Table Characteristics of Study Participants included into the Analysis and of Non-participants. ICCC-3, International Classification of Childhood Cancer, 3rd Edition; CNS, central nervous system. ^a)^ p-values calculated from *t*-test (age) and from chi-square-tests (gender, deceased at time of study, diagnoses) comparing participants and non-participants. ^b)^ Including ICCC-3 main groups: (6), (7), (10), (11) and (14).
(DOCX)

## Abbreviations

CAM: complementary and alternative medicine
IKOM: Institute of Complementary Medicine
SCCR: Swiss Childhood Cancer Registry
CNS: central nervous system
LCH: Langerhans cell histiocytosis
NCCIH: National Center for Complementary and Integrative Health of the USA
CTX: chemotherapy
RTX: radiotherapy
ICCC-3: International Classification of Childhood Cancer, 3rd Edition
CI: confidence intervals
