# Supplementary material for: Use of Complementary and Alternative Medicine in Children with Cancer: A Study at a Swiss University Hospital
Source: PLoS One. 2015 Dec 22;10(12):e0145787. doi: 10.1371/journal.pone.0145787 (PMC4687920; doi:10.1371/journal.pone.0145787)
Supplement: S1 Table — ICCC-3, International Classification of Childhood Cancer, 3rd Edition; CNS, central nervous system. a) p-values calculated from t-test (age) and from chi-square-tests (gender, deceased at time of study, diagnoses) comparing participants and non-participants. b) Including ICCC-3 main groups: (6), (7), (10), (11) and (14). (DOCX) [file pone.0145787.s001.docx]

**S1 Table. Characteristics of Study Participants included into the Analysis and of Non-participants**

|  | Participants  (N=133) | | Non-participants (N=164) | | p-value ^a)^ |
| --- | --- | --- | --- | --- | --- |
|  | n | (%) | n | (%) |  |
| *Age of patients at diagnosis* |  |  |  |  | 0.140 |
| 0 – 4 years | 60 | (45) | 57 | (35) |  |
| 5 – 9 years | 29 | (22) | 43 | (26) |  |
| 10 – 13 years | 28 | (21) | 33 | (20) |  |
| 14 – 18 years | 16 | (12) | 31 | (19) |  |
| *Gender of patients* |  |  |  |  | 0.290 |
| Female | 63 | (47) | 67 | (41) |  |
| Male | 70 | (53) | 97 | (59) |  |
| *Patients deceased at time of study* | 15 | (11) | 41 | (25) | 0.004 |
| *Diagnoses (ICCC-3 main group)* |  |  |  |  | 0.947 |
| (1) Leukemia, myeloproliferative diseases and myelodysplastic syndrome | 41 | (31) | 52 | (32) |  |
| (2) Lymphoma and reticuloendothelial neoplasms | 21 | (16) | 19 | (12) |  |
| (3) CNS tumors and miscellaneous intracranial and intraspinal neoplasms | 32 | (24) | 41 | (25) |  |
| (4) Neuroblastoma and other peripheral nervous cell tumors | 9 | (7) | 7 | (4) |  |
| (8) Malignant bone tumors | 7 | (5) | 8 | (5) |  |
| (9) Soft tissue and other extraosseous sarcomas | 8 | (6) | 13 | (8) |  |
| Other diagnoses ^b)^ | 15 | (11) | 24 | (15) |  |
